# High Temperature Metal Hydrides as Heat Storage Materials for Solar and Related Applications

**DOI:** 10.3390/ijms10010325

**Published:** 2009-01-15

**Authors:** Michael Felderhoff, Borislav Bogdanović

**Affiliations:** Max-Planck Institut für Kohlenforschung, Kaiser-Wilhelm-Platz 1, 45470 Mülheim /Ruhr, Germany

**Keywords:** Hydrogen storage, heat storage, magnesium hydride, Mg_2_FeH_6_

## Abstract

For the continuous production of electricity with solar heat power plants the storage of heat at a temperature level around 400 °C is essential. High temperature metal hydrides offer high heat storage capacities around this temperature. Based on Mg-compounds, these hydrides are in principle low-cost materials with excellent cycling stability. Relevant properties of these hydrides and their possible applications as heat storage materials are described.

## 1. Introduction

The paper on hand deals with a chemical-based method for thermal solar energy storage. Materials which are appropriate for this purpose are chemical compounds of metals, metal alloys or intermetallic compounds and hydrogen known as *metal hydrides* (MH_n_, Equation 1). The majority of metals, metal alloys and intermetallic compounds react directly with gaseous hydrogen (Equation 1 from left to right) to form metal hydrides in an exothermal way, that is, with release of heat (Δ*H*). If the formation of a metal hydride under certain conditions is a reversible reaction ( 
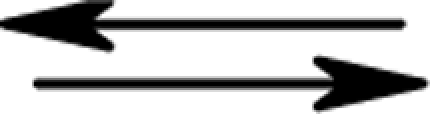
), then the metal hydride can by a heat supply upon decomposition into the metal and hydrogen. Upon thermal dissociation of a metal hydride by heat absorption (Equation 1, from right to left), the thermal energy is transformed into chemical energy. Expressed in another way: thermal energy can be reversibly stored as the heat of reaction of reversible chemical reactions. Among the methods for heat storage, sensible and thermochemical heat storage, the thermochemical method offers in general the higher amount of energy stored per mass of storage material (Table 1) [1, 2].

The special feature of metal hydrides as reversible heat storage systems is that upon their thermal dissociation the liberated hydrogen is simultaneously a fuel with the highest known gravimetric energy density (usually expressed in MJ·kg^−1^). The gravimetric energy density of hydrogen [1, 3] is about three times higher than that of gasoline. Hydrogen in liquid form is therefore used as a rocket fuel. A further remarkable feature of hydrogen as a fuel is that upon its combustion at high temperatures in air [4] as well as upon “cold combustion” in low temperature fuel cells, only pure water is produced. Thus, from ecological standpoint hydrogen is an ideally clean, albeit a secondary energy carrier. The last attribute means that hydrogen has first to be produced under consumption of primary energy sources which are at present primarily fossile fuels or, otherwise, nuclear energy (via water electrolysis) or, in the future, the expected solar energy. From the above statements it follows that the reversible metal hydride/metal systems (Equation 1 ) are to be considered as energy carriers in a double sense - namely as heat and as hydrogen storage systems.


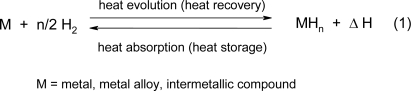


In the heat storage application, the heat of reaction liberated in the hydride formation process serves as useful heat. Thereby hydrogen is confined in a closed system and is preserved during a great number of heat storage cycles. In the other case – as a hydrogen store – the hydrogen liberated from the hydride is present in an open system, and after displacement from the hydride it is irreversibly burned to water.

## 2. The principle of heat and hydrogen storage by the MgH_2_/Mg system

**Figure 1.**
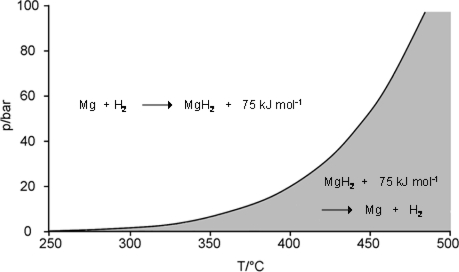
Dissociation pressure curve of MgH_2_.

The magnesium hydride/magnesium system is particularly suitable for both purposes, because in MgH_2_ the reversible hydrogen storage capacity is the highest among reversible binary hydrides (7.6 wt.%) and because upon the reaction of magnesium with hydrogen (Equation 2 ) a relatively large amount of heat (75 kJ·mol^−1^ H_2_, (0.9 kWh kg-1Mg) at a temperature level between 200 and 500 °C is set free. Herewith an extremely important region for heat storage becomes available, since according to the Carnot principle, heat energy at this level can be transformed with high efficiency into other energy forms like mechanical energy and hence in electricity. We are dealing here mainly with the application of magnesium hydride as a heat storage medium. The MgH_2_/Mg system (Equation 2), however, can also be applied for hydrogen storage. Upon gradually heating up of MgH_2_ in a closed system the compound will dissociate giving Mg and H_2_ and the hydrogen pressure above the remaining MgH_2_ will increase exponentially following the van’t Hoff law (Figure 1).


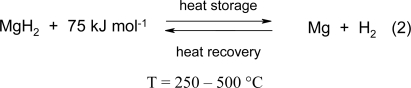


The knowledge of the H_2_ dissociation (equilibrium) pressure as a function of temperature is of fundamental importance for the operation of the MgH_2_ heat and hydrogen storage system. At a given H_2_ pressure the course of the curve in Figure 1 determines which minimal temperature that is definitely necessary in order to liberate the hydrogen from MgH_2_ under a given H_2_ pressure (f.i. 450 °C for 40 bar of H_2_ pressure); and conversely, upon charging of Mg with hydrogen under given pressure conditions the temperature of the store will correspond to the hydrogen charging pressure. Thus, by choice of the hydrogen pressure it is possible to determine at which temperature level the stored heat will be delivered. The diagram in the Figure 1 shows additionally under which temperature and pressure conditions the system will absorb or desorb heat (shaded part - heat absorption; clear part - heat desorption). Although the diagram in the Figure 1 indicates under which conditions the reaction can go in one or in the other direction, it does not give any information if at all and how rapidly the reaction will take place. In the early [5] and in the more recent literature [6] it is often stated that the reaction of magnesium with hydrogen is notoriously sluggish and therefore hardly useful for heat storage [5]. For an optimal thermochemical heat storage material it is required that the chemical reaction be much more rapid than the rate of heat transport, so that the dynamics of the system will depend mainly on the rate of the heat transport. Additionally, it is required that the reaction in both directions is any times repeatable and that the material shows no sign of fatigue. For accelleration of chemical reactions, apart from increase of temperature, and the effect of the microstructure of particles [6], the general answer is catalysis.

**Figure 2.**
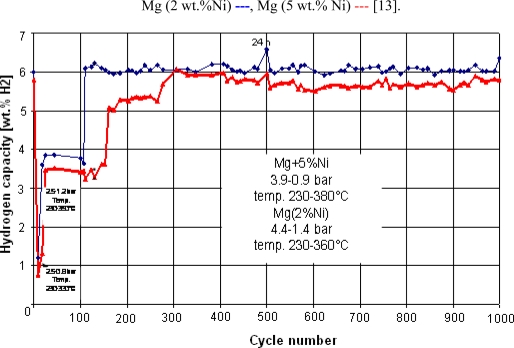
1000 cycle test of Ni-doped MgH_2_, time for a full cycle 3 h.

At the beginning of our research work on metal hydrides there was the experimental finding that the reaction of magnesium with hydrogen in solution can be catalyzed [7]. During the past years the research in this field has led to MgH_2_/Mg materials which fulfill the above mentioned criteria: satisfactory kinetics combined with stability upon cyclic hydrogen charging and discharging. The first method for the preparation of the so-called “active MgH_2_/Mg materials” (Equation 3) [1, 8] consists in treatment of commercial Mg powder (f.i. 270 mesh) with hydrogen in an organic solvent (i.e. toluene) in the presence of a small amount of the soluble organometallic nickel complex bis(cyclooctadiene-1,5-Ni(0) [9] at ambient temperature and normal pressure. The Ni-complex is hereby instantaneously hydrogenated to cyclooctane and metallic nickel and the latter, in a fine dispersion, is precipitated on the surface of the Mg particles and acts as a catalyst for hydrogenation and dehydrogenation of the former. The second method [8] consists of intimately mixing of the Mg powder with 4–10 wt.% of fine commercial Ni powder. The activity of the material prepared according to the second method is somewhat lower than that of the first method. Cycle stability of the “standard materials” is presented in Ref. [8] (Figures 4, 5 and 9 therein). The Mg powders doped with Ni in these ways and fabricated several times on a 10 kg scale were shown in numerous tests to be suitable materials for heat and hydrogen storage and were employed by us and by others in several storage installation prototypes [1, 8, 10–12], three of which are presented in Section 3. Already after the first hydrogenation the materials are fully active and remain active during hundreds of hydrogen charging-discharging cycles. A more recent 1,000 cycle test using both materials is shown in Figure 2 [13]. Some restrictions at high temperatures are discussed further in Section 4.


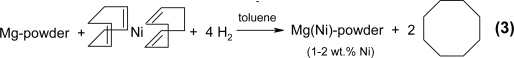


**Figure 3.**
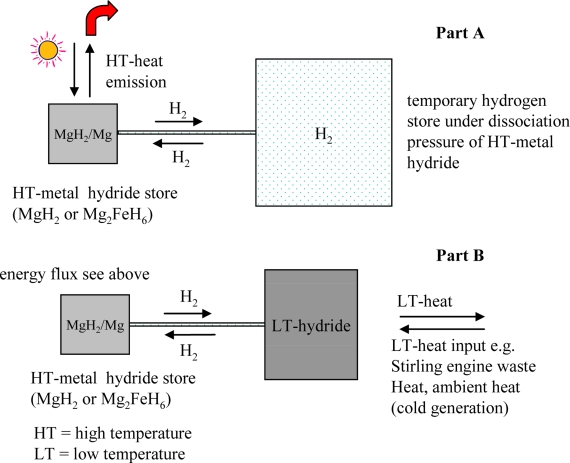
Schematical representation of construction of MgH_2_/Mg heat stores. Part A: temporary storage of hydrogen in a pressure container; part B: temporary storage of hydrogen in a low-temperature metal hydride.

As already mentioned (Equation 2), hydrogen is liberated in the endothermic process of feeding heat (75 kJ mol^−1^ Mg) to MgH_2_, which until the reverse process of heat recovery must be temporarily stored. A heat storage system based on MgH_2_-Mg (Figure 3) has to be constructed as a closed system consisting of a MgH_2_ pressure container and an additional pressure container for the temporary storage of the displaced hydrogen (Part A). As schematically represented (Figure 3, Part A), the magnesium hydride container can be designed so to be much smaller than the pressure container for gaseous hydrogen, because of the much higher hydrogen concentration in the form of magnesium hydride. In the form of MgH_2_ hydrogen attains a concentration corresponding to a hydrogen pressure of 700 – 800 bar. Up to ~150 °C MgH_2_ has only a very low dissociation pressure, which means that hydrogen storage in this form is comparably a secure form for hydrogen storage.

In comparison to the first version (Figure 3, Part A), a much more compact construction can and has been realized, when for the temporary storage of hydrogen in place of a pressure container is applied an at low temperature hydrogen absorbing alloy (Figure 3, Part B) which with hydrogen forms a so-called low temperature (LT) metal hydride. A LT hydride is typically a hydride on Fe-Ti or La-Ni-basis [14] or, in future possibly on alanate basis [15].

Using LT hydrides for temporary storage of hydrogen, hydrogen is available at ambient or even below ambient tremperature. Apart from the compact construction, such a heat store (Figure 3, Part B) includes possibilities which are of great importance for the rational use of solar and other energy sources:

- absorption of hydrogen by the LT-alloy produces LT-heat (f.i. 30–70 °C) which is typically ≈ 1/3 of the MgH_2_ heat amount (75 kJ mol^−1^); this waste heat can be used for example for warm water supply;
- the desorption of hydrogen from the side of the LT hydride takes place with withdrawal of heat from the surroundings, that is with production of cold, that can be used for production of ice or for climatisation.
- altogether a closed circular system of two (or more) conjoined reversible metal hydride systems which operate at different temperature levels represent a so-called hydride chemical heat pump, which is applicable for different types of heat and energy transformations [16].

## 3. Experimental results concerning the MgH_2_-Mg system

### *3.1. A process steam generator* [17, 18]

In the following three installations which are designed for different fields of application and which have been realized as laboratory prototypes are described. These were primarily engineering tasks which were tackled at first by engineers by mathematical modelling (simulations) and calculations.

**Figure 4.**
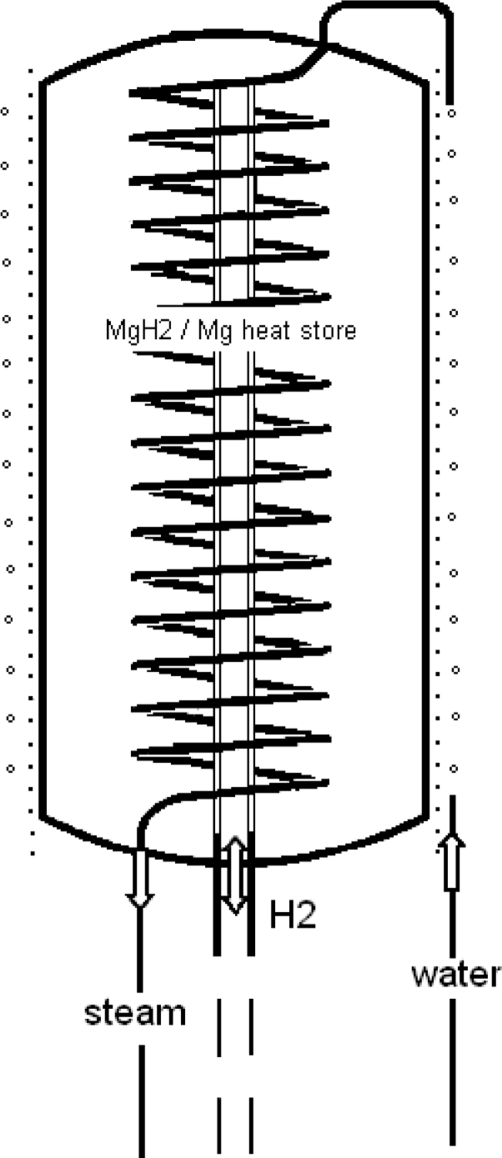
The process steam generator based on MgH_2_ [17, 18].

Unlike engineers, chemists prefer an experimental approach. In this sense the first experimental heat accumulator on magnesium hydride basis was built and tested in our institute. The facility for the production of the process steam was installed in the pilot plant of the institute and was in operation during 1½ years. In this connection the heat transfer problems within the hydride bed (heat pipe effect) was also investigated. The process steam generator is schematically represented in Figure 4, the isolated cylindric steel pressure vessel is filled with 14.5 kg of Ni-doped Mg-powder.

In the center of the vessel is installed a sintered metal tube for supply and removal of hydrogen. Around the vessel is wound a steel tube which is connected to a source for pressurized water. The tube is further lead into the inner of the vessel where it is helically embedded in the MgH_2_/Mg bulk. Hydrogenation of the Ni-doped storage material is carried out directly in the pressure vessel. For heat desorption the pressure vessel is electrically heated from outside, resulting in MgH_2_ dissociation. The hydrogen which escapes is temporary stored in a bunch of six commercial hydrogen pressure containers. As soon as the heating is interrupted and the temperature of the heat accumulator begins to decrease the hydrogen flows back from the pressure containers to the Mg/MgH_2_ vessel where the HT heat is regenerated and produces in the spiral tube superheated water vapor. Some technical data about the heat store unit are given in the Table 2.

The heat accumulator had the ability to produce 4 kW of heat, up to 90% capacity at the same temperature level. A coupled unit of a MgH_2_/Mg heat store with a hydrogen pressure container (Figure 3, Part A) can also serve as a heat buffer for intermittent HT heat sources.

### *3.2. A model of a thermochemical solar power plant* [1, 8, 11, 13, 19, 20]

**Figure 5.**
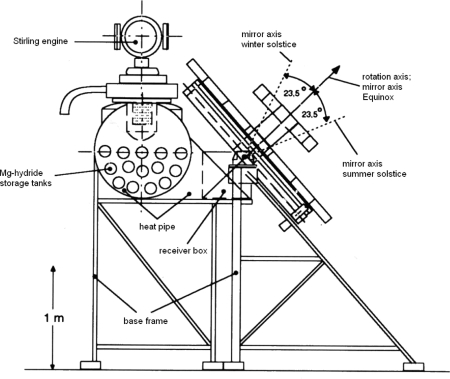
Cross section of the model of the solar power station, with MgH_2_ heat storage units and fix-focus solar concentrator.

The MgH_2_/Mg-heat accumulator, first used to demonstrate the feasibility for production of “process vapor”, is clearly applicable for storage of industrial heat and at this temperature level (350–450 °C) of solar heat in particular. According to the “solar concept”, the combination of a solar-thermal power generator with a thermochemical MgH_2_ heat accumulator should enable generation of power also in the absence of sunshine, that is after sundown and during the passage of clouds. Within the frame of a publicly sponsored joint project between the both Max-Planck-Institutes in Mülheim, the IKE Institute of the Stuttgart University and the HTC Solar Company in Lörrach a first model of a small solar power station was built. Figure 5 shows a cross section of the installation. The main components were a “fixed focus concentrator” for solar radiation [11], a cavity radiation receiver, a heat pipe system for heat transfer, a Stirling engine, a MgH_2_ store, a hydrogen pressure or LT hydride store. For supply of the whole installation with solar heat a solar mirror collector with 6.5 m² area of a light weight type construction was built. The concentrated solar heat is used to drive the Stirling engine and produce power. Simultaneously, a more or less part of the solar heat is absorbed by the MgH_2_ heat store and the released hydrogen streams in the pressure container or, alternatively, in the LT hydride store. In periods without or weak solar irradiation the MgH_2_/Mg system cools down and the hydrogen flows back to the MgH_2_ store and produces there high temperature heat and via Stirling engine electric power. The model of the solar power plant was tested under laboratory [10] and in limited extent also under solar test field conditions [11] and in this way the ability of the system to function was established. Shortcomings of the installation were realized and the measures to address them proposed.

### *3.3. A solar cooking- and cooling-device* [12, 19]

As a third example for the magnesium hydride application for heat storage and transformation a laboratory model of a solar cooking-and-cooling device was built. The device consists of a MgH_2_-heat store unit filled with 4.4 kg (~3 KWh heat) of the Ni-doped Mg-material and equipped with a cooking plate. The unit is linked to a LT metal hydride store unit with ~30 kg of a LT metall alloy (MmNi_4.22_Fe_0.78_) coupled to a refrigerator and a water reservoir. As a cooling cabinet a commercial ice-box of 113 L capacity is used. The solar mirror in this case has an area of 2.5 m^2^ and is made out of a fibrous plastic material.

During solar irradiation hours solar heat is concentrated by means of the fixed-focus concentrator and directly used for cooking. Simultaneously, hydrogen from the MgH_2_-store is desorbed and absorbed in the LT alloy, thus performing the task of heat storage. The LT heat of 30–40 °C is absorbed by a water reservoir. In order to be able to cook in the absence of solar irradiation the hydrogen is allowed to stream in the reverse direction, so that the heat of MgH_2_ formation provides heat for cooking. During the hydrogen desorption from the LT hydride, the desorption heat is removed from the surroundings and the resulting cold with the help of a water-glycol circle system is transferred to the ice-box in which ice is produced.

A complete cycle of an experimentally performed cooking- and cooling process is described in the following. At the beginning of such a cycle hydrogen is located in the LT hydride store. Upon opening of the valve between the two stores, the hydrogen streams to the HT-store resulting in a heat evolution of around 300 °C which remains during 5–6 h at this level and is usable for cooking. At the same time the LTH store itself cools down to -10 °C; ice is formed in the ice-box and the temperature there remains at about 0 °C. In this time 0.9 KWh of cold are produced and 35% of this amount are allotted for the production of ice. After a rest of 7 h during the night, during which the hydrogen supply is disconnected, through solar irradiation, at 350–450 °C, hydrogen is displaced from MgH_2_ and absorbed in the LT alloy with formation of warm water (30–40 °C). The device is now ready for a new cooking-cooling cycle. Laboratory tests were satisfactory, but solar (field) tests have not yet been done. As operational areas can be envisaged regions with a lot of sunshine but without energy infrastructure. Without any consumption of fuels or electric power it should be possible to cook and to refrigerate food at any day or night time.

## 4. Ni-doped versus undoped MgH_2_-Mg materials for high temperature heat or hydrogen storage [21]

**Figure 6.**
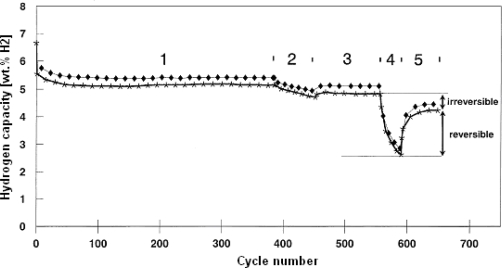
A 650 cycle test performed with mechanically Ni-doped (4 wt.%) 270 mesh Mg powder, demonstrating reversible and irreversible hydrogen capacity losses under severe conditions; —*— and —♦—, 45 and 135 min hydrogenation times, respectively [21].

The design and construction of the above described appliances for energy and heat storage on MgH_2_/Mg basis are more or less tasks for engineers. For chemists there still remains the challenge of improving the properties of metal hydride storage materials and to develop new ones. So, for instance, the question arises which is the highest application temperature level for functioning of MgH_2_/Mg heat storage system. The question is of great importance, because according to the Carnot principle the efficiency of heat engines – be they small Stirling engines or steam turbines – increases with the temperature level of the fed heat. In order to remain with our example: the process steam of 500 °C is more efficient for the production of power than the same heat quantity at 400 °C or lower. If under this aspect we look again at temperature dependence of the MgH_2_ dissociation pressure (Figure 1), it can be seen that at the temperature of, f.i. 500 °C, the hydrogen pressure in the system is around 100 bar. Results of a 650 cycle test with mechanically Ni-doped Mg-powder (4 wt.%) under severe conditions are shown in Figure 6 [21].

Under a high dehydrogenation temperature (section 2, 480 °C) the hydrogen capacity and with it, the amount of stored heat, decreases. An investigation of this phenomenon showed that a part of these capacity losses can be recovered under milder conditions (Sections 2/3, 4/5, “reversible capacity losses”). A part of the capacity losses, however, is permanent (Sections 1/3, 3/5, “irreversible capacity losses”). Additionally, it could be shown that capacity losses upon cyclisation are higher with Ni-doped material than with undoped Mg-powder. The consequence is therefore that for heat storage at a high temperature level (> ~440 °C), it is of advantage to use plain Mg-powder in place of a Ni-doped one. The cause for this fall-off in performance at higher temperatures, as we presently know it, is a morphological one – namely the sintering of Mg particles at temperatures above 450 °C, which is more pronounced in a two (Mg-Ni) than in an one component system (Mg) [21].

## 5. The Mg-Fe-H system and its potential for thermochemical thermal energy storage [22]

### 5.1. Mg_2_FeH_6_ as reversible ternary hydride system

A way out of the dilemma discussed in Section 4 was to look for alternative metal hydride/metal systems in which such sintering phenomena do not occur. To overcome this problem magnesium iron hydride Mg_2_FeH_6_ can be used for heat storage. The compound was discovered and the structure elucidated in 1984 by Yvon *et al*. after heating of 2:1 mixtures of 2 Mg and Fe powders at 450 °C under 20–120 bar H_2_ pressure [23].

**Figure 7.**
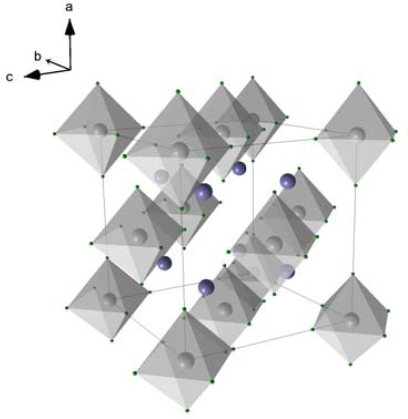
Crystal unit cell of Mg_2_FeH_6;_ Mg-atoms are shown in blue, Fe-atoms are located in the centers of the octahedrons.

**Figure 8.**
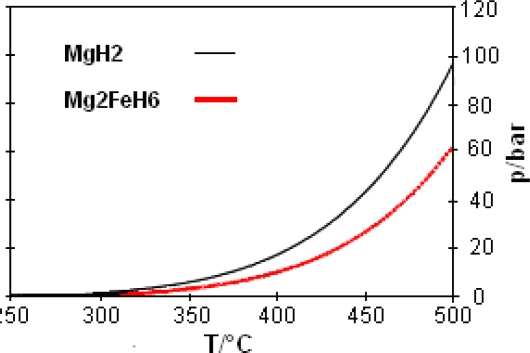
Temperature dependence of the dissociation pressure of Mg_2_FeH_6_ in comparision with that of MgH_2_ [22].

As it can be seen in Figure 7, Mg_2_FeH_6_ is composed of Mg^2+^ cations and FeH_6_^4-^ anions in the form of octahedrons. In the center of the octahedrons is located a Fe atom, which is surrounded by six covalently bound H atoms. Alternatively to the high pressure method, Mg_2_FeH_6_ can be synthesized by reactive mechanical alloying of Fe and Mg elemental powders in a hydrogen atmosphere at room temperature [24–26]. The gravimetric hydrogen content of Mg_2_FeH_6_ is 5.5 wt.%. The volumetric hydrogen density reaches 150 kg m^−3^ which is double of the density of liquid hydrogen (70 kg m^−3^). The advantage of Mg_2_FeH_6_ as a heat storage material in comparison to MgH_2_ (Table 3) is its higher stability and hence a lower hydrogen dissociation pressure (Figure 8). So, for example, the dissociation pressure of Mg_2_FeH_6_ at 500 °C (66 bar) - is about 1/3 lower than that of MgH_2_ at the same temperature (100 bar). This gives for the construction of pressure containers for Mg_2_FeH_6_ an advantage versus containers for MgH_2_.

**Figure 9.**
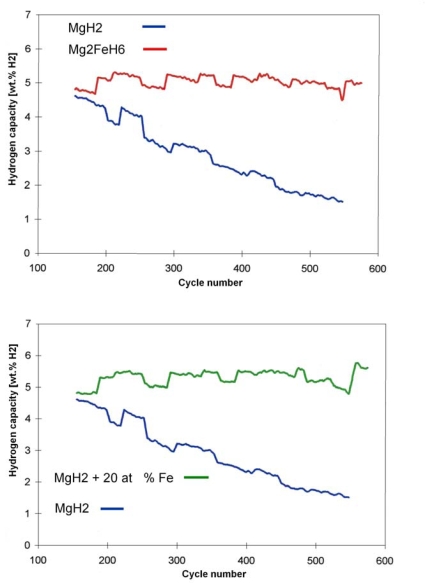
Hydrogen storage capacity of MgH_2_, Mg_2_FeH_6_ (top) and of the mixed Mg_2_FeH_6_-MgH_2_ system [22]. (Conditions for re/dehydrogenations: 482/533 °C, 80/86 bar, 1.5/1.5 h).

Numerous cycle tests done with the Mg-Fe-H system prove that the system is superior under high temperature and pressure conditions with respect to cycle stability in comparison to the pure MgH_2_-Mg system (Figure 9, top) [22]. While the latter system under different HT conditions already looses capacity after 200–250 cycles (due to sintering), the Mg-Fe-H-systems, under comparable conditions, show no signs of loss of capacity and reaction rates even after 600 or more cycles. Even under very severe conditions (510–590 °C/138–149 bar) cycle stabilities with a capacity of more than 5 wt.% H_2_ are achieved. Interestingly, the improved cycle stability has been shown to exist also using less than stoichiometric amounts of Fe with respect to Mg (Figure 9, bottom). In view of the possible technical application of these systems for heat storage in future especially of solar heat in the temperature region around 500 °C or higher, this finding appears to be of importance [22]. However, further investigation of cycling properties of the mixed MgH_2_/Mg – Mg_2_FeH_6_/2Mg Fe hydride sytems is necessary.

### 5.2. Electron microscopy investigations of the Mg_2_FeH_6_ materials

**Figure 10.**
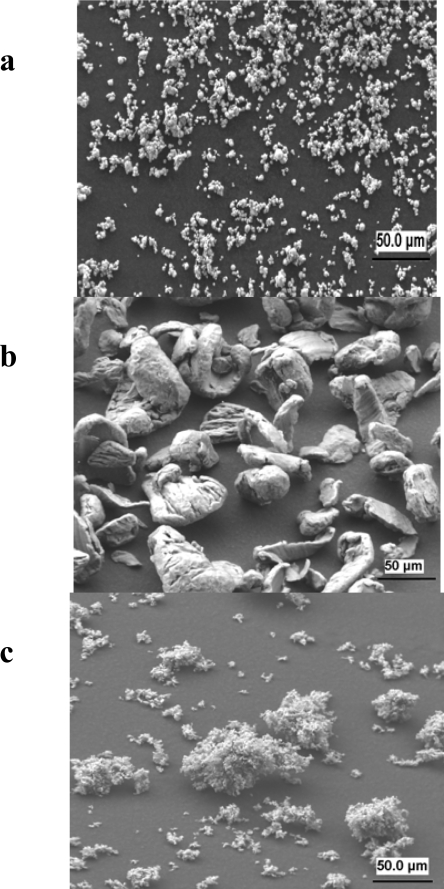
Raster electron images from top to the bottom: a) Fe-metal, b) Mg-flakes, c) Mg_2_FeH_6_ after 600 cycles.

**Figure 11.**
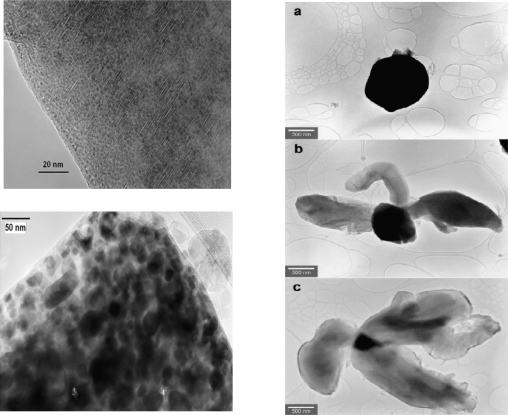
Left: HR-TEM-pictures of Mg_2_FeH_6_ (top) and dehydrogenated material (2Mg + Fe) (bottom); right: TEM micrographs of different steps of Mg_2_FeH_6_ formation are recorded. Dark regions of the particles are Fe regions and the lighter consist of Mg_2_FeH_6_. (a) Initial stage of the Mg_2_FeH_6_ formation; (b) vermicular excresence of Mg_2_FeH_6_ out of the surface of an iron seed; (c) final stage of the Mg_2_FeH_6_ formation [22].

The cause for the improvement of thermochemical storage properties of the Mg-Fe-H system with respect to the MgH_2_ system could be partially elucidated via electron microscopy investigations on Mg-Fe-H samples in the hydrogenated and dehydrogenated state. Raster electron- microscopy images of the starting Fe and Mg powders (Fe 5–10 μm and Mg particles 50–100 μm) and of Mg_2_FeH_6_ after 600 hydrogenation/dehydrogenation cycles at high temperatures are shown in Figures 10a–c. A high resolution transmission electron microscopy (TEM) micrograph of Mg_2_FeH_6_ is reproduced in Figure 11 (top). At 1:500,000 magnification one can already perceive the atomic net plains of the crystalline magnesium-iron hydride. The material is perfectly homogeneous. On the lower part of the Figure (Figure 11, bottom) can be seen a TEM photo of the dehydrogenated material: in this case black and colorless regions can be discerned. By energy dispersive X-ray (EDX) analyses it could be proved that the black regions are almost pure iron and the colorless transparent ones are pure magnesium. Upon each dehydrogenation the two metals separate from each other.

The size of the regions are in the range of 20–50 nm. Apparently, due to this extremely fine dispersion it is possible that after each hydrogenation step the homogeneous Mg_2_FeH_6_ phase is formed. In the hydrogenation step a transfer of (probably) magnesium atoms must take place. With the help of TEM it was possible to identify different stages of Mg_2_FeH_6_ formation from Fe, Mg and H_2_ and which takes place on the surface of Fe grains (Figure 11); for details see Ref. [22]. It appears plausible that this special circumstance - the separation of metals in two immiscible phases upon dehydrogenation and the merging to one phase upon hydrogenation - counteracts agglomeration of particles and thus, is one of the probable causes of cyclic stability.

## 6. A picture of a thermochemical storage of solar heat on the MgH_2_/Mg basis

### 6.1. Comparision of the MgH_2_/Mg system with the existing heat storage system of Andasol 1

**Figure 12.**
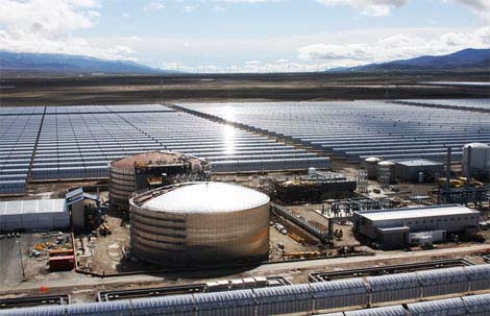
50 MW Solar power plant Andasol 1 with solar thermal energy storage system [27], photo with permission from Solar Millennium.

Andasol 1 is a solar thermal power plant located in Andalucia (Spain) with a 50 MW steam turbine for electricity production. To run the plant during the dark a heat storage system is installed for additional 7.5 h electricity production at night. The storage system consists of two insulated storage tanks each 14 m high and 36 m in diameter (Figure 12). They are filled with 28,000 tons of a molten salt mixture of sodium and potassium nitrate. The tanks are kept at different temperatures, the cold tank at 260 °C and the hot tank at 390 °C. To charge heat, the “cold” molten salt picks up heat in a heat exchanger from the 400 °C hot oil of the solar field and stores in the hot tank. For discharging the process is reversed [27]. The overall storable heat amount in this system is 1,000 MWh [28].

As mentioned in Section 2 the amount of heat released upon reaction of 1 kg of Mg with H_2_ is 0.9 kWh. Thus it can be roughly estimated that an amount of about 1,100 tons of Mg-metal would be able to store the same amount of heat as the Andasol storage system. In addition to the storage of the Mg/MgH_2_ mixture, a second container is necessary for the temporary storage of the hydrogen released during the dissociation of MgH_2_ (heat absorption Figure 3, Part A). The most simple option is the storage of hydrogen gas under own dissociation pressure of MgH_2_. As an example we assume here working temperatures of the heat storage between 370 and 400 °C. At 370 °C the dissociation pressure of MgH_2_ is 10 bar and at 400 °C roughly 20 bar (Figure 1) [29]. In this pressure and temperature ranges, a simple calculation shows [30], a volume of 109,000 m^3^ of H_2_ gas resulting from decomposition of MgH_2_ must be stored. This sounds a lot, but, as shown below, gas storage of this order of magnitude has been standard gas technology for more than hundred years.

### 6.2. Options for the storage of large volumes of hydrogen gas

#### 6.2.1. Hydrogen gas storage in containers

Natural gas storage under elevated pressures in spherical gas tanks is a widely used method. A system with a diameter up to 40 m and a maximum gas pressure of roughly 10 bar for natural gas has been constructed in Wuppertal, Germany and is in use since the 1950’s. The volume of this storage vessel is 55,000 m^3^. Pipe containers are another simple method for the storage of natural gas up to pressures of 100 bar. A similar construction can also be used for the storage of hydrogen.

#### 6.2.2. Hydrogen storage in gas pipelines

Large volumes of H_2_ can be stored in existing hydrogen pipelines. A hydrogen pipeline can possibly be situated near a thermal solar power plant.

#### 6.2.3. Hydrogen gas storage in geological formations

The storage of natural gas in underground salt caverns is also a widely used method to overcome fluctations in the demand for the gas [31]. Recently, several studies have shown that underground caverns can be used for the storage of hydrogen under elevated pressures and with extremely low leak rates [32, 33]. Underground hydrogen storage in salt caverns is situated, f.i. in Teesside, Great Britain. The salt caverns are in a depth of 350 m with an overall volume of 70,000 m^3^. The hydrogen gas is under a constant pressure of 45 bar, which is the result of brine replacement. These values are close to those of a conceptual storage system based on a MgH_2_/Mg storage system for a 50 MW solar thermal power plant. On condition is that a suitable geological formation can be found close to the power plant, but underground hydrogen storage option appears to be a cheap and simple storage method for hydrogen released from MgH_2_.

## 7. Conclusions

In the face of todays’ urgent climate, energy and economical problems we are at present witnessing intensified planning and building of thermal solar power plants [34]. To enable the plants to function overnight storage of HT heat is necessary, which in practice usually involves storage of sensible heat by means of molten salts. As chemists we propose that, as presented in Section 6, the “thermochemical option” based on HT metal hydrides should also be taken into consideration.

On the basis of the present short overview one can easily recognize that in the research field of metal hydrides as energy storage systems both at high and low temperatures there remains yet a lot to be discovered. Currently the search for low temperature reversible hydride systems with higher than at present known H_2_ storage capacity, as required for heat pumps and production of cold, but first and foremost as hydrogen storage materials for fuel-cell driven cars [15, 35], seems especially worthy of efforts at the moment.

## Figures and Tables

**Table 1.** Frequently used solid and liquid materials for the storage of sensible heat [1].

	M.p. [°C]	Temperature range [°C]	ρ [g cm^−3^]	*c*[kJ kg^−1^K^−1^]
Water	0	0 – 100	0.98 [a]	4.19
Thermo-oil	−70 / −10	up to 350 [b]	0.87 [a]	2.1
53 KNO_3_/40 NaNO_2_/7 NaNO_3_[c]	142	up to 450	1.85 [a]	1.3
Na	98		0.84 [d]	1.26
Cast iron	1150 – 1300		7.2 [d]	0.54
Aluminium	660		2.7 [d]	0.92
Fire clay			2.1–2.6 [d]	1.0
Al_2_O_3_	1700		3.0 [d]	1.0
MgO	1700		3.0 [d]	1.0
Stone			1.9–2.6 [d]	0.8–0.9
Concrete				0.9

[a] verage density in the temperature range given,

[b] some thermooils can be used up to 390 °C,

[c] wt.%,

[d] at room temperature.

**Table 2.** Technical Data of the MgH_2_-Heat Store Unit [17, 18].

Amount of storage material (Ni-doped Mg)	14.5 kg
Volume of the pressure vessel	19.4 dm^3^
Weight of the pressure vessel	26 kg
Bulk density	0.75 g cm^−3^
Maximum operational pressure	50 bar
Maximum operational temperature	450 °C
Total weight	40 kg
Amount of stored heat / total weight	0.25 kWh kg^−1^
Maximum power output	4 kW
Amount of water to be vaporized at the power output of 4 kW	6 kg h^−1^
Maximum steam temperature	ca. 400 °C
Maximum steam pressure	40 bar

**Table 3.** Comparision of important values for heat storage using MgH_2_ and Mg_2_FeH_6_ [22].

	MgH_2_	Mg_2_FeH_6_
Reaction enthaply ΔH [kJ mol^−1^]	74	77.4
Hydrogen content calc. [wt.%]	7.66	5.47
Hydrogen content expt. [wt.%]	6	5
Theoretical density of the hydride [g cm^−3^]	1.42	2.74
Experimentally realized density [g cm^−3^]	0.8	1.22
Heat storage density (calc. weight) [kJ kg^−1^]	2,814	2,106
Heat storage density (expt. weight) [kJ kg^−1^]	2,204	1,921
Heat storage density (calc. volume) [kJ dm^−3^]	3,996	5,768
Heat storage density (expt. volume) [kJ dm^−3^]	1,763	2,344

## References

[1] Bogdanović B, Ritter A, Spliethoff B (1990). Active MgH_2_-Mg systems for reversible chemical energy storage. Angew. Chem. Int. Ed.

[2] Beckmann G, Gilli PV (1984). Thermal Energy Storage.

[3] Buchner H (1982). Energiespeicherung in Metallhydriden.

[4] The formation of NO_x_ in application of H_2_ for engines with internal combustion can be practically eliminated using lean gas mixtures (λ λ 2); taken from [3], p. 126.

[5] Rummel W (1978). Heat storage in magnesium-hydrogen system. Siemens Forsch. Entwicklungsber.

[6] Zaluska A, Zaluski B, Ström-Olsen JO (1999). Nanocrystalline magnesium for hydrogen storage. J. Alloys Comp.

[7] Bogdanović B, Liao S, Schwickardi M, Sikorsky P, Spliethoff B (1980). Catalytic synthesis of magnesium hydride under mild conditions. Angew. Chem. Int. Ed. Engl.

[8] Bogdanović B, Hartwig Th, Spliethoff B (1993). The development, testing and optimization of energy storage materials based on the MgH_2_-Mg system. Int. J. Hydrogen Energy.

[9] Bogdanović B, Kröner M, Wilke G (1966). Olefin-Komplexe des Nickels (0). Liebigs Ann. Chem.

[10] Wierse M, Groll M, Veziroglu TN, Winter CJ, Baselt JP, Kreysa G (1996). Development, construction and testing of a thermo-chemical energy store on the MgH_2_ basis for solar power plants and other energy supplying systems (in German), a joint project of the German BMFT, 0328939 B, 1995; Wierse, M.; Groll, M. System performance of a solar thermal power station with a thermochemical energy storage.

[11] Kleinwächter J, Mitzel M (1996).

[12] Steiner D, Wierse M, Groll M, Veziroglu TN, Winter CJ, Baselt JP, Kreysa G (1996). Development of a solar cooking/cooling unit with a thermochemical energy store based on metal hydrides.

[13] Reiser A, Bogdanović B, Schlichte K (2000). The application og Mg-based metal hydrides as heat energy storage systems. Int. J. Hydrogen Energy.

[14] Bowman RC, Fultz B (2002). Metallic hydrides I: Hydrogen storage and other gas-phase applications. MRS Bulletin.

[15] Schüth F, Bogdanović B, Felderhoff M (2004). Light metal hydrides and complex hydrides for hydrogen storage. Chem. Commun.

[16] Dantzer P, Wipf H (1997). Metal-hydride technology: A critical review. Hydrogen in Metals III, Topics in Applied Physics..

[17] Bogdanović B, Ritter A, Spliethoff B, Straßburger K (1995). A process steam generator based on the high temperature magnesium hydride/magnesium heat storage system. Int. J. Hydrogen Energy.

[18] Straßburger K Wärmetransformation und thermische Energiespeicherung durch das aktive MgH_2_/Mg-System Thesis, University Essen Essen, Germany 1999

[19] Bogdanović B, Spliethoff B, Ritter A (1989). The magnesium hydride system for heat storage and cooling. Ztschr. Physik. Chem. Neue Folge.

[20] Bogdanović B, Ritter A, Spliethoff B, Straßburger K (1992).

[21] Bogdanović B, Hofmann H, Neuy A, Reiser A, Schlichte K, Spliethoff B, Wessel S (1999). Ni-doped versus undoped Mg-MgH_2_ materials for high temperature heat or hydrogen storage. J Alloys Compd.

[22] Bogdanović B, Reiser A, Schlichte K, Spliethoff B, Tesche B (2002). Thermodynamics and dynamics of the Mg-Fe H system and its potential for thermochemical thermal energy storage. J. Alloys Comp.

[23] Didisheim JJ, Zolliker P, Yvon K, Fischer P, Schefer J, Gubelmann M, Williams AF (1984). Dimagnesium Iron(II) Hydride, Mg_2_FeH_6_, Containing Octahedral FeH_6_^4-^ Anions. Inorg. Chem.

[24] Huot J, Boily S, Akiba E, Schulz R (1998). Direct synthesis of Mg_2_FeH_6_ by mechanical alloying. J. Alloys Compd.

[25] Gennari FC, Castro FJ, Andrade Gamboa JJ (2002). Synthesis of Mg_2_FeH_6_ by reactive mechanical alloying: formation and decomposition properties. J. Alloys Compd.

[26] Varin RA, Li S, Calka A, Wexler D (2004). Formation and environmental stability of nanocrystalline and amorphous hydrides in the 2Mg-Fe mixture processed by controlled reactive mechanical alloying (CRMA). J. Alloys Compd.

[27] Fairly P Largest Solar Thermal Power Plant to Start Up http://www.spectrum.ieee.org/oct08/6851, accessed October 2008.

[28] http://www.nrel.gov/csp/troughnet/pdfs/2007/martin_andasol_pictures_storage.pdf, accessed March 2007.

[29] Bogdanović B, Bohmhammel K, Christ B, Reiser A, Schlichte K, Vehlen K, Wolf U (1999). Thermodynamic investigation of the magnesium hydrogen system. J. Alloys Compd.

[30] The amount of 1,100 tons of Mg, when converted into MgH_2_, would upon dissociation deliver 1.09 × 10^6^ Nm^3^ of H_2_ or 109,000 m^3^ at 10 bar pressure. Thus, for temporary storage of hydrogen a storage tank of 109,000 m^3^ capacity is necessary. We envisage that at the beginning of a heat storage cycle the tank is filled with 10 bar of hydrogen. Upon heating of the storage unit filled with 1,100 tons of MgH_2_ from 370 to 400 °C, the unit, upon complete dissociation of MgH_2_ would release 109,000 m^3^ of hydrogen, which when introduced into the storage tank would result in a pressure increase from 10 to 20 bar (Figure 1). During night the heat store unit will cool down and the hydrogen will flow back to the heat store, producing there 1,000 MWh of heat at the 400 to 370 °C temperature level. The pressure in the system will drop from 20 to 10 bar, thus restoring the initial state. It should be mentioned that the described system is self-controlled, depending only of the amount of heat out- or input to the storing unit. Certainly, the presented is a very crude outline of a heat storage cycle, since during the cycle ubiquitous heat losses have to be taken into account. On the other hand, by assuming higher than at presently used working temperatures and pressures for the heat storage cycle, much smaller volumes for the hydrogen storage tank can be calculated.

[31] Sedlacek R (1999). Underground Gas Storage in Europe. Erdöl, Erdgas, Kohle.

[32] Leighty W (2008). Running the world on renewables: Hydrogen transmission pipelines and firming geologic storage. Int. J. Energy Res.

[33] Crotogino F Hamelmann R Wasserstoff-Speicherung in Salzkavernen zur Glättung des Windstromangebotes. 14. Symposium zur Nutzung regenerativer Energiequellen und Wasserstofftechnik, Stralsund, Germany, Nov. 2007 Energieperspektiven 2 2008, http://www.ipp.mpg.de/ippcms/ep/ausgaben/ep200802/bilder/wasserstoff_speicher, accessed 2008.

[34] http://www.solarpaces.org/News/Projects/_projects.htm, accessed 2008.

[35] Züttel A, Borgschulte A, Schlapbach L (2008). Hydrogen as a Future Energy Carrier.

